# In Vitro Autonomous Construction of the Flagellar Axial Structure in Inverted Membrane Vesicles

**DOI:** 10.3390/biom10010126

**Published:** 2020-01-11

**Authors:** Hiroyuki Terashima, Chinatsu Tatsumi, Akihiro Kawamoto, Keiichi Namba, Tohru Minamino, Katsumi Imada

**Affiliations:** 1 Division of Biological Science, Graduate School of Science, Nagoya University, Furo-cho, Chikusa-Ku, Nagoya 464-8602, Japan; terashima.hiroyuki@h.mbox.nagoya-u.ac.jp; 2 Department of Macromolecular Science, Graduate School of Science, Osaka University, 1-1 Machikaneyama, Toyonaka, Osaka 560-0043, Japan; tatsumic14@gmail.com; 3 Graduate School of Frontier Biosciences, Osaka University, 1-3 Yamadaoka, Suita, Osaka 565-0871, Japan; kawamoto@protein.osaka-u.ac.jp (A.K.); keiichi@fbs.osaka-u.ac.jp (K.N.); tohru@fbs.osaka-u.ac.jp (T.M.); 4 RIKEN SPring-8 Center and Center for Biosystems Dynamic Research, 1-3 Yamadaoka, Suita, Osaka 565-0871, Japan; 5 JEOL YOKOGUSHI Research Alliance Laboratories, Osaka University, 1-3 Yamadaoka, Suita, Osaka 565-0871, Japan

**Keywords:** bacterial flagellum, type III secretion system, flagellar specific export apparatus, inverted membrane vesicle, in vitro reconstitution, flagellar filament, *Salmonella typhimurium*

## Abstract

The bacterial flagellum is a filamentous organelle extending from the cell surface. The axial structure of the flagellum consists of the rod, hook, junction, filament, and cap. The axial structure is formed by axial component proteins exported via a specific protein export apparatus in a well-regulated manner. Although previous studies have revealed the outline of the flagellar construction process, the mechanism of axial structure formation, including axial protein export, is still obscure due to difficulties in direct observation of protein export and assembly in vivo. We recently developed an in vitro flagellar protein transport assay system using inverted membrane vesicles (IMVs) and succeeded in reproducing the early stage of flagellar assembly. However, the late stage of the flagellar formation process remained to be examined in the IMVs. In this study, we showed that the filament-type proteins are transported into the IMVs to produce the filament on the hook inside the IMVs. Furthermore, we provide direct evidence that coordinated flagellar protein export and assembly can occur at the post-translational level. These results indicate that the ordered construction of the entire flagellar structure can be regulated by only the interactions between the protein export apparatus, the export substrate proteins, and their cognate chaperones.

## 1. Introduction

**Figure 1:**
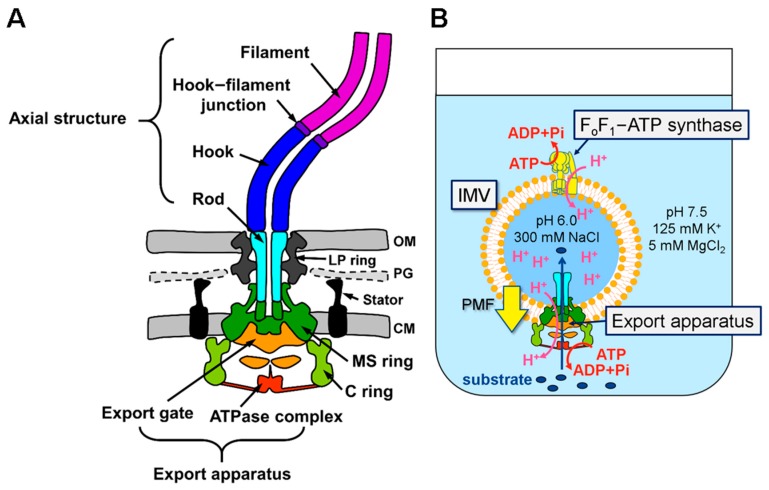
Schematic drawing of the *Salmonella* flagellum (**A**) and the in vitro transport assay system using the inverted membrane vesicles (IMVs) (**B**). (A) The sub-structures of the *Salmonella* flagellum are represented in the following colors: the transmembrane export gate, orange; the cytoplasmic ATPase complex, red; the MS-ring, green; the C-ring, light green; the rod, cyan; the hook, blue; the hook-filament junction, purple; the filament, magenta; the LP ring, gray; the stator, black. The core of the export apparatus consists of the export gate and the ATPase complex. The filamentous part composed of the rod, the hook, the hook–filament junction and the filament are called the flagellar axial structure. CM, the cytoplasmic membrane; PG, the peptidoglycan layer; OM, the outer membrane. (B) To apply the initial PMF to the IMVs, the IMVs were filled with 300 mM NaCl at pH 6.0 and suspended in solution with 125 mM K^+^ and 5 mM MgCl_2_ at pH 7.5. The export substrates, ATP-Mg^2+^, the FliH_2_/FliI complex, and FliJ were added to the assay mixture. To maintain PMF across the inverted membrane, endogenous F_o_F_1_-ATP synthase pumps proton into the IMVs by ATP hydrolysis energy. PMF and ATP hydrolysis energy generated by FliI ATPase drives the substrate protein transport into the IMVs.

The bacterial flagellum is a tubular organelle extending out from the cell surface, and is rotated by a nano-scale rotary motor embedded in the cytoplasmic membrane. Flagellar construction starts with the assembly of the basal structure containing the MS-ring, the C-ring, and the flagellar protein export apparatus in the cytoplasmic membrane, followed by assembly of the filamentous axial structure composed of the rod, the hook, the hook–filament junction, the filament, and the filament cap on the basal structure (Figure 1A) [1,2,3]. A certain copy number of component proteins assemble into each substructure in a specific order [1]. The axial proteins are transported via the flagellar export apparatus, which belongs to the type III secretion system family, into the central channel of the growing tubular axial structure and diffuse through it to the distal end, where they are incorporated into the structure [4,5]. The export apparatus consists of a transmembrane export gate composed of FlhA, FlhB, FliP, FliQ, and FliR, and a cytoplasmic ATPase complex composed of FliH, FliI, and FliJ [1,2,3]. The MS-ring and the C-ring function as a housing for the export gate and a sorting platform for the cytoplasmic ATPase complex, respectively [6,7].

Flagellar construction is a well-regulated process, in which the expression and export of flagellar axial proteins are coupled with the assembly state of the flagellum. The flagellar axial proteins are classified into two groups by the substrate-recognition mode of the flagellar protein export apparatus: one is the rod/hook-type substrate class, responsible for the assembly of the rod and hook structures, and the other is the filament-type substrate class, required for the construction of the hook–filament junction, the filament, and the filament cap. Before completion of the hook (the early stage of flagellar formation), only the rod/hook-type proteins are allowed to be exported, and the export of filament-type proteins is suppressed [8,9]. After the length of the hook has reached approximately 55 nm, the export of rod/hook-type proteins is stopped, and the filament-type proteins begin to be exported (the late stage of flagellar formation) [10,11,12]. Thus, the switching of the substrate specificity of the flagellar protein export apparatus from the rod/hook-type to the filament-type proteins is a crucial step in regulating the flagellar construction, as well as in controlling the hook length. The hook length is monitored by a secreted molecular ruler protein, FliK [13,14]. FliK is a rod/hook-type protein and is infrequently exported during hook assembly to measure the hook length using its N-terminal disordered region [15,16]. When the hook length is too short, FliK is secreted out into the extracellular media. When the hook length reaches approximately 55 nm, the C-terminal domain of FliK binds to FlhB, one of the export gate component proteins, to induce conformational changes of FlhB and FlhA to switch the substrate specificity of the export apparatus [17,18,19,20,21,22]. The filament-type proteins form a complex with their specific cognate chaperones, which prevent premature aggregation and/or proteolysis of their cognates and help them associate with the flagellar protein export apparatus in the cytoplasm [23,24,25]. The flagellar chaperones not only facilitate the docking of their cognate filament-type proteins to the protein export apparatus, but also regulate the export order through the interactions with FlhA, FliI, and FliJ [1,2]. Moreover, the flagellar chaperones are multifunctional proteins able to control the production of flagellar proteins as well as delivering their cognates to the export apparatus [26,27,28].

Genetic and biochemical studies have revealed that there are several morphological checkpoints involved in coordination of flagellar protein export and assembly, not only at the gene expression level but also at the post-translational level. However, the molecular mechanism of each process, including protein export, is still unclear because of difficulties in direct observation of protein export and assembly in vivo. To overcome this problem, we recently established an in vitro flagellar protein transport assay system using inverted membrane vesicles (IMVs) to quantitatively control and measure protein export and monitor the flagellar assembly process (Figure 1B) [29,30]. We demonstrated that the flagellar protein export apparatus in the IMVs maintains the export function for rod/hook-type proteins at a level similar to that in a living cell, and that ATP hydrolysis by FliI ATPase dramatically accelerates the export of rod/hook-type proteins. Proton-motive force (PMF) across the cytoplasmic membrane is a primary driving force for flagellar protein export [31,32,33], and the energy derived from ATP hydrolysis by FliI ATPase is thought to be required for the activation of the PMF-driven export gate complex [34]. However, our study with IMVs revealed that the energy of ATP hydrolysis is able to drive protein export even when PMF is absent [29]. Moreover, we successfully reproduced not only hook formation inside the IMVs, but also hook length control. However, we have not yet shown the export of the filament-type proteins via the switched export apparatus in the IMVs. Since the filament-type proteins require their specific cognate chaperones for efficient export [2], the protein export mechanism, including its regulation, would be expected to be different from that for the rod/hook-type proteins.

Here, we showed that the filament-type substrates (FlgK, FlgL, FliC, FliD) in complex with their cognate chaperones are transported into the interior of the IMVs after completion of the hook. These transported filament-type proteins assemble into the filament at the tip of the hook. Moreover, even when the hook-type proteins (FlgD, FlgE, FliK) and filament-type proteins in complex with substrate-specific flagellar chaperones were simultaneously added to the in vitro transport assay solutions, these proteins autonomously and sequentially assembled into the normal flagellar structure, indicating that the coupling of flagellar gene expression with assembly is not really required for well-ordered flagellar formation.

## 2. Materials and Methods

### 2.1. Bacteria Strains and Plasmids

Bacterial strains and plasmids used are listed in Table 1. The primers used in this study are shown in Supplementary Table S1. *Salmonella* and *Escherichia coli* cells were cultured in LB broth (1% (*w*/*v*) bactotryptone, 0.5% (*w*/*v*) yeast extract, 0.5% (*w*/*v*) NaCl). Chloramphenicol was added to a final concentration of 30 µg/mL. Ampicillin was added to a final concentration of 50 µg/mL.

### 2.2. Preparation of Inverted Membrane Vesicles

The inverted membrane vesicles (IMVs) were prepared according to the method described by Terashima et al. [29]. We prepared the IMVs from a *Salmonella* cell strain STH001 (∆flhB, ∆flgD, and ∆fliT) harboring plasmid pITH103 (wild-type flhB and flhDC). The cells were cultured at 37 °C for 9 h, inoculated into 1 L of LB broth with 1/100 dilution and cultured at 30 °C for 1 h. L-arabinose was then added at the final concentration of 0.02% (*w*/*v*) for induction of FlhB and FlhD_4_/FlhC_2_, and the culture was continued at 18 °C for 12–16 h until OD_600_ reached around 1.5. The cells were collected by centrifugation and suspended in 75 mL of sucrose solution (10 mM Tris-HCl pH 8.0, 0.75 M sucrose), to which was added 22.5 mg of lysozyme powder. Next, 150 mL of 1.5 mM EDTA-NaOH pH 8.0 was poured into the cell suspension on ice to form spheroplasts. The cell suspension was stirred on ice for 1 h. The spheroplasts were collected at 5000× *g* for 10 min and suspended in 25 mL solution A (20 mM MES-NaOH pH 6.0, 300 mM NaCl) with a half tablet of protease inhibitor cocktail (cOmplete EDTA-free, Roche). In order to produce IMVs, the cell suspension was passed through high-pressure cell homogenizer (STANSTED) at 90 MPa. After centrifugation at 20,000× *g* for 10 min to remove cell debris, the membrane vesicles were precipitated by ultra-centrifugation at 100,000× *g* for 1 h. The crude IMVs were suspended in 1 mL of solution A and separated by sucrose density-gradient centrifugation (60% (*w*/*w*) 5 mL/50% (*w*/*w*) 9 mL/45% (*w*/*w*) 9 mL/40% (*w*/*w*) 6 mL stepwise gradient in the Beckman ultra-clear tube) at 60,000× *g* (SW32 Ti rotor: Beckman, Brea, CA, USA) for 16 h. A brown-colored layer fraction containing the IMVs was recovered, and the IMVs were precipitated by ultra-centrifugation at 100,000× *g* for 1 h. The precipitant was suspended in 900 μL of solution A. The suspension was divided into 300 µL aliquots, frozen with liquid nitrogen and stored at −80 °C. The frozen stock was thawed and filtered with a 0.8 µm polycarbonate filter (Nuclepore membrane PC MB 19 0.8U, GE healthcare, Chicago, IL, USA). The filtered solution was loaded onto a Sephadex G-50 fine column (GE healthcare) and eluted with solution B (125 mM KCl, 20 mM Tris-HCl pH 7.5). The concentration of IMVs was adjusted to give OD 600 nm = 0.1.

### 2.3. Protein Purification

FlgD, FlgE, FliK, the FliH_2_/FliI complex, and FliJ were purified according to the method described by Terashima et al. [29]. The FliC/FliS, FliD/FliT, FlgK/FlgN, or FlgL/FlgN complex was expressed in *E. coli* BL21(DE3) cells from the plasmids pITH110, pITH113, pITH117, or pITH118, respectively. The cells harboring pITH110 or pITH117 were inoculated directly from the colonies onto the agar plate into LB broth and incubated overnight at 30 °C to express the proteins by leak-expression from pTrc99a-based plasmids. The cells harboring pITH113 or pITH118 were grown at 30 °C for overnight, inoculated into fresh LB broth with 1/100 dilution and cultured at 30 °C until the optical density at 600 nm reached 0.5–0.8. IPTG was then added to the final concentration of 0.1 mM, and the culture was continued at 18 °C for overnight. The cells expressing FliC/FliS, FliD/FliT, FlgK/FlgN, or FlgL/FlgN were suspended in cell-suspend solution (50 mM Tris-HCl pH 8.0, 500 mM NaCl) containing protease inhibitor cocktail (cOmplete EDTA-free, Roche, Rotkreuz, Switzerland), and disrupted by sonication. After removal of cell debris by low-speed centrifugation followed by filtration with a 0.45 µm cellulose acetate membrane filter device, the cell lysate was loaded to a HisTrap HP column (GE healthcare), and the hexahistidine-tagged proteins were then eluted by linear imidazole gradient. To chop off the hexahistidine tag, protein solutions were incubated with thrombin (GE healthcare) at room temperature for 3 h and then passed through HisTrap HP again to remove hexahistidine-tag-retained proteins. Finally, the protein solution was purified using a Superdex 200 column (GE healthcare) equilibrated with external solution (20 mM Tris-HCl pH 7.5, 125 mM KCl). The purity of the purified proteins was examined by SDS-PAGE and Coomassie Brilliant Blue staining (Supplementary Figure S1).

### 2.4. Transport Assay

Transport assay of the hook-type proteins was carried out as previously described [29]. Transport assay of the filament-type proteins were carried out using the IMVs after hook formation by the transport reaction. FlgD, FlgE, FliK, FliJ, and the FliH_2_/FliI complex were added into the IMV solution (20 mM Tris-HCl, pH 7.5, 125 mM KCl, 5 mM MgCl_2_, and 1 mM dithiothreitol (DTT)) at the final concentration of 4 µM, 4 µM 4 µM, 0.25 µM, and 1.5 µM, respectively. After addition of ATP with a final concentration of 5 mM, the mixture was incubated at 37 °C for 1 h to form the hook in the IMVs and then was ultra-centrifuged at 100,000× *g* for 30 min. The precipitant was washed by external solution and the solution was removed. The precipitant was resuspended in 100 µL of external solution and used for the filament-type protein transport assay. The IMV solution was incubated with the filament-type proteins (2 µM), FliJ (0.25 µM), the FliH_2_/FliI complex (1.5 µM), MgCl_2_ (5 mM), and ATP (5 mM) at 37 °C for 2 h. The proteins transported in the IMVs were detected by immunoblotting.

### 2.5. Purification of the Hook–Basal Body from IMV

The hook–basal body complexes were purified according to the method described by Terashima et al. [29]. The IMV was solubilized by 0.1% (*v*/*v*) Triton X-100. The suspension was ultra-centrifuged at 150,000× g for 30 min. The filament–hook–basal body complex was precipitated and suspended in TET solution (10 mM Tris-HCl pH 8.0, 1 mM EDTA, 0.1% (*v*/*v*) Triton X100). In order to completely dissolve the membrane, the suspension was mixed with 1 mL of alkali solution (10% (*w*/*v*) sucrose, 0.1% (*v*/*v*) Triton X100, 0.1 M KCl, adjusted to pH 11.0 by KOH). The suspension was layered on 1 mL of 35% (*w*/*v*) sucrose solution prepared by dissolving sucrose in TET solution in an ultra-centrifuge tube. After incubation on ice for 30 min, the suspension was ultra-centrifuged at 38,000 rpm (Beckman TLA100.3 rotor) for 30 min to precipitate the filament–hook–basal body. The precipitates were suspended in TET solution and then observed by electron microscopy.

### 2.6. Dynamic Light Scattering

A total 12 uL of purified IMVs in solution B (OD 600 nm = 0.1) was injected into a low-volume quartz cuvette and measured using Zetasizer μV (Malvern Panalytical, Malvern, UK). The scattering data were analyzed using Zetasizer software (Malvern Panalytical).

### 2.7. Negative-Staining Electron Microscopy

Sample solutions were applied to carbon-coated copper grids and negatively stained with 2.0% (*w*/*v*) phosphotungstic acid or 2.0% (*w*/*v*) uranyl acetate. Images were observed with a JEM-1010 transmission electron microscope (JEOL, Tokyo, Japan) operating at 100 kV using a BIOSCAN model 792 CCD camera, a JEM-1011 transmission electron microscope (JEOL, Tokyo, Japan) operating at 100 kV using a TVIPS TemCam-F114 CCD camera or a TemCam-F415 CCD camera, or a JEM-2010 transmission electron microscope (JEOL, Tokyo, Japan) operating at 200 kV using a Orius SC200D model 833 CCD camera (Gatan, Pleasanton, CA, USA).

## 3. Results

### 3.1. In Vitro Protein Transport of the Filament-Type Proteins into the IMVs

We previously showed that FlgE, FlgD, and FliK, which belong to the rod/hook-type substrate class, are transported into the IMVs through the flagellar protein export apparatus to form the hook on the endogenous rod in the basal body inside the IMVs [29]. The transported FlgE molecules were assembled into the hook with the help of the FlgD cap, and the hook length was controlled to approximately 55 nm by the addition of FliK to the assay solutions. These results suggested that the FliK molecule alone is sufficient the hook length control, and terminates the export of the rod/hook-type proteins in the in vitro protein transport assay system [29]. However, we had not examined whether FliK actually induces the switching of substrate specificity of the flagellar protein export apparatus from the rod/hook-type to the filament-type proteins. We therefore studied the export of the filament-type proteins into the IMVs after the termination of rod/hook-type protein export triggered by FliK.

**Figure 2:**
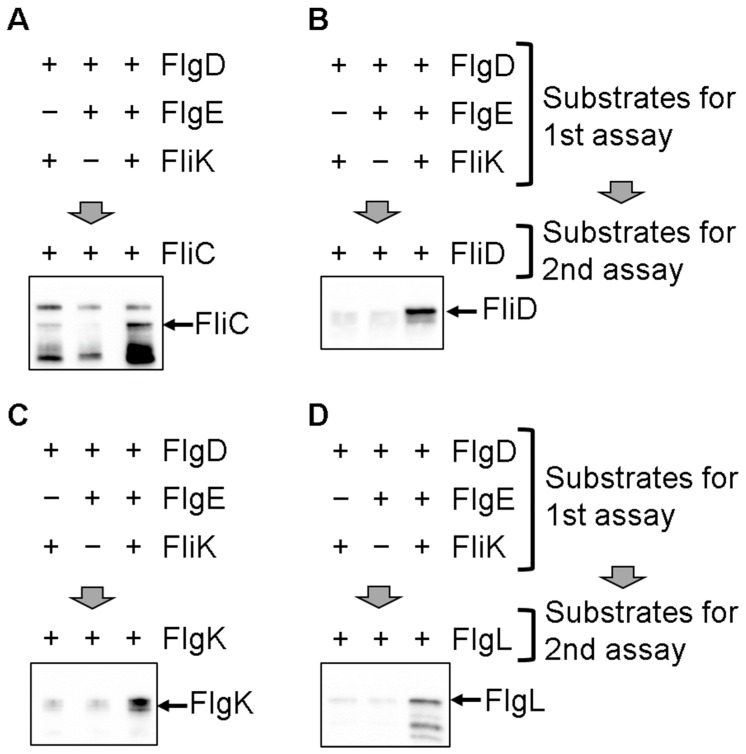
Hook formation is essential for the filament-type protein transport. The first reaction mixture for hook formation contained FlgD, the FliH_2_/FliI complex, FliJ, and ATP at final concentrations of 4 µM, 1.5 µM 0.25 µM, and 5 mM, respectively. FlgE and FliK were added to the mixture at the final concentration of 4 µM, respectively. The first transport reaction was carried out at 37 °C for 1 h. After the reaction, the IMVs were precipitated by ultra-centrifugation (100,000× *g*, 30 min) and used for the second transport reaction to transport the filament-type substrates. Re-suspended IMVs were mixed in the second transport reaction mixture containing 1.5 µM of the FliH_2_/FliI complex, 0.25 µM of FliJ, 5 mM of ATP–Mg, and 2 µM of the filament-type substrate–chaperone complex: FliC/FliS (**A**), FliD/FliT (**B**), FlgK/FlgN (**C**), or FlgL/FlgN (**D**).

We first incubated IMVs with the substrate proteins required for hook formation (FlgD, FlgE, and FliK), the cytoplasmic ATPase complex proteins (the FliH_2_/FliI complex and FliJ), ATP, and Mg^2+^ for 1 h at 37 °C to form the hook in the IMVs, as previously described [29]. The IMVs were precipitated by ultra-centrifugation and then used for in vitro transport assays for the filament-type proteins. The IMVs with the hook–basal bodies were suspended into transport assay mixtures containing the FlgN/FlgK, FlgN/FlgL, FliS/FliC, and FliT/FliD chaperone–substrate complexes and the components of the cytoplasmic ATPase complex. The mixtures were then incubated for 2 h at 37 °C after adding ATP and Mg^2+^. FlgK, FlgL, FliC, and FliD were transported into the IMVs, but were not transported in the absence of either FliK or FlgE (Figure 2). These results indicate that both FliK and the presence of the completed hook structure are required for the export of the filament-type proteins into the IMVs. Therefore, we suggest that purified FliK alone triggers switching of the substrate specificity of the flagellar protein export apparatus upon completion of hook assembly.

### 3.2. Effect of the FliH_2_/FliI Complex on Filament-Type Protein Export

**Figure 3:**
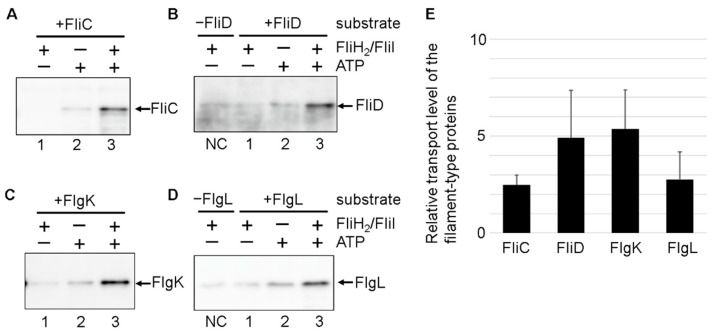
In vitro protein transport of the filament-type substrates into the IMVs. IMVs after completion of the hook were mixed with reaction mixture containing 0.25 µM of FliJ and 2 µM of the filament-type substrate–chaperone complex: FliC/FliS (**A**), FliD/FliT (**B**), FlgK/FlgN (**C**), and FlgL/FlgN (**D**). The transport assay was conducted with (+) or without (−) the FliH_2_/FliI complex (1.5 µM) and ATP (5 mM). The bands in Lane 1 in (**B**,**C**) are cross-reacting bands because similar bands were detected in the assay not containing the substrates (Lane NC in (**B**,**C**)). (**E**) The transport levels of the filament-type protein relative to those without the FliH_2_/FliI complex. The immunoblot band intensity was measured using Image J software. The relative transport level was calculated by dividing the band intensity of Lane 3 by that of Lane 2 after subtraction of that of Lane 1 (without ATP). Data from three independent experiments were averaged. Error bar represents standard deviation.

The FliH_2_/FliI complex greatly facilitates the export of rod/hook-type proteins such as FlgD and FlgE. The addition of the FliH_2_/FliI complex to the final concentration of 1.5 μM to the assay solution increased the relative FlgD transport level 20-fold [29]. Therefore, we analyzed the impact of the FliH_2_/FliI complex on filament-type protein export. The transport levels of the filament-type substrates were significantly increased by adding 1.5 μM FliH_2_/FliI complex, similarly to FlgD export (Figure 3). These results suggest that the FliH_2_/FliI complex facilitates the export of filament-type proteins. 

### 3.3. Filament Structure Formation on the Hook in the IMVs

**Figure 4:**
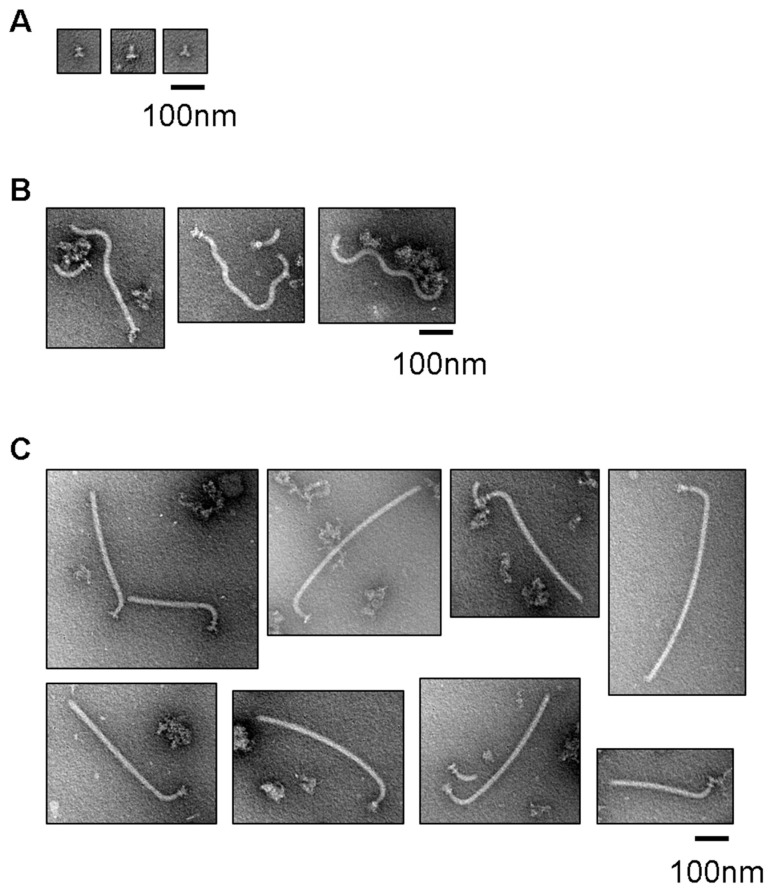
Negative-staining electron micrographs of the basal bodies after transport assay of the filament-type proteins purified from the IMVs. The basal bodies after transport assay using the IMVs prepared after first reaction with FlgD and FliK but without FlgE (**A**), with FlgD and FlgE but without FliK (**B**), and with FlgD, FlgE, and FliK (**C**). The concentrations of FlgD, FlgE, FliK, FliH_2_/FliI complex, FliJ, and ATP–Mg in the first reaction mixture were 4 µM, 4 µM, 4 µM, 1.5 µM, 0.25 µM, and 5 mM, respectively. The concentrations of FliC/FliS, FliD/FliT, FlgK/FlgN, and FlgL/FlgN in the transport assay mixture for the filament-type proteins were 1 µM each and those of the FliH_2_/FliI complex, FliJ, and ATP–Mg were the same as in the first reaction mixture.

We next examined whether the filament-type proteins exported into the IMVs formed the filament at the tip of the hook. We prepared the IMVs possessing the hook as shown above, and suspended them in an assay mixture containing the FlgN/FlgK, FlgN/FlgL, FliS/FliC, and FliT/FliD complexes (1 µM each), the FliH_2_/FliI complex (1.5 µM), FliJ (0.25 µM), ATP (5 mM), and Mg^2+^ (5 mM). After incubation for 2 h at 37 °C, the IMVs were collected, washed and solubilized with detergent. Then the flagellar hook–basal bodies were precipitated by ultra-centrifugation, negatively stained with phosphotungstic acid, and observed by electron microscopy. We found the filament attached on the hook–basal body just like the filament–hook–basal body complex purified from *Salmonella* cells, indicating that the filament-type proteins and their cognate chaperones are sufficient to form the filament (Figure 4C) and no other soluble factor is needed for the protein export apparatus to coordinate the export of filament-type proteins with filament formation at the tip of the completed hook. The filament was not formed when either FliK or FlgE was absent in the reaction mixtures (Figure 4A,B), supporting the idea that the filament is formed only after completion of the hook. These results suggest that filament formation in the IMVs proceeded in the same way as in vivo.

We measured the length of the filament by electron microscopy. The filament length was widely distributed from 36 to 890 nm, with an average length of 287 nm (±166 nm), suggesting that the filament length was not controlled (Supplementary Figure S2). The filaments formed in the IMVs were much shorter than those extended from the *Salmonella* cell body [34], implying that the filament growth was restricted by the inner space of the IMVs. Dynamic light scattering (DLS) measurement showed that the Z-average hydrodynamic radius of IMVs was 275 nm ± 121, which was consistent with the average lengths of the filaments formed in the IMVs.

### 3.4. Effect of Uncoupling of Flagellar Expression with Assembly on the Entire Assembly Process of the Hook–Filament Complex in the IMVs

**Figure 5:**
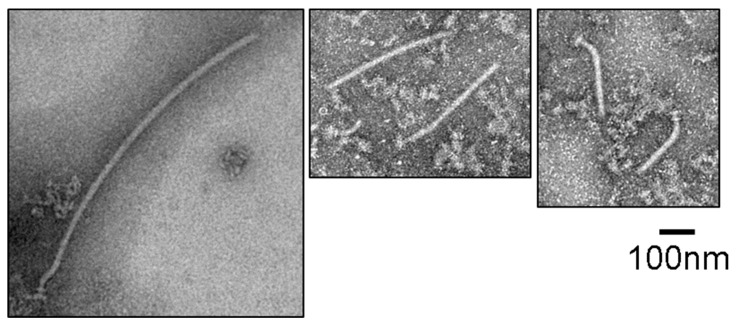
Autonomous hook–filament complex formation in the IMV. Negative-staining electron micrographs of the basal bodies after transport assay in the reaction mixture containing all the export proteins, their cognate chaperones, the export ATPase complex components, ATP, and Mg^2+^.

We also examined whether the entire process of hook–filament construction proceeded inside the IMVs without adding any soluble components other than those that were used in the above experiment. We prepared a transport assay mixture containing FlgD, FlgE, FliK (1 µM each), the filament-type proteins in complex with their cognate chaperones (1 µM each), the ATPase complex proteins (0.25 µM FliJ and 1.5 µM FliH_2_/FliI), ATP (5 mM), and Mg^2+^ (5 mM). The IMVs were incubated in the mixture for 4 h at 37 °C, collected by ultra-centrifugation, washed, and solubilized with detergent. The flagellar axial structure was then precipitated by ultra-centrifugation and observed by electron microscopy. The hook–filament structure was formed on the basal body, indicating that the entire process of hook–filament formation proceeded inside the IMVs with only the protein components preexistent in the IMVs and those added in the external solution (Figure 5 and Supplementary Figure S3). This suggests that the coupling of gene expression with the flagellar assembly process is not essential for ordered flagellar formation, although it may be important for efficient construction of the flagellum.

## 4. Discussion

The flagellar hook–filament complex has been reconstructed in solution using purified FlgK, FlgL, FliC, and FliD monomers, and purified hook fragments as a template [38]. However, this is just spontaneous self-assembly and is completely different from in vivo flagellar assembly, in which the assembly process is coupled with flagellar axial protein export. To understand protein export and the following assembly process, we developed an in vitro protein transport assay system that can reproduce the export and assembly process of the bacterial flagellar axial proteins that occur in living cells [29]. Our system uses IMVs and purified proteins. In a series of studies, we have demonstrated that the flagellar protein export apparatus in the IMVs preserves the transport activity of flagellar axial proteins. The hook–filament complex was successfully constructed from FlgD (hook-capping protein), FlgE (hook protein), FlgK (first junction protein), FlgL (second junction protein), FliC (filament protein, flagellin), and FliD (filament-capping protein) transported into the interior of the IMVs. Moreover, the important functions and events, such as hook length control and substrate specificity switch, were nicely reproduced in the IMVs. Thus, the IMV-based system is a powerful tool for investigating the mechanisms of flagellar protein export and assembly.

Our in vitro experiments using the IMVs clearly revealed that the substrate specificity switching of the flagellar protein export apparatus is triggered by FliK alone, and that no other factors are needed for the switching event. When the hook was preassembled inside the IMVs, the filament was formed on the hook without adding any proteins other than the filament components, their cognate chaperones, and the ATPase complex proteins. Moreover, the hook–filament structures were constructed in the IMVs even when all the necessary proteins and factors were added to the external solution at once. These results indicate that coordinated flagellar protein export is not obligately linked to the regulated flagellar gene expression, although they are coupled to each other in the living cell, probably for a more efficient assembly process. 

Our results also suggest that the entire flagellar construction process, including the ordered protein export and assembly, is regulated by only the interactions of the PMF-driven export gate with the cytoplasmic ATPase complex, the export substrates, and export chaperones, without the involvement of any other factors. Although the ordered assembly of flagellar axial proteins is achieved in principle by a template-structure driven mechanism based on specific protein–protein interactions, an ordered protein export that could be caused by different affinities of export substrates for the export apparatus may play a role in increasing the assembly efficiency. In fact, the binding affinity of the substrate–chaperone complexes for the export apparatus correlates with the export order of the filament-type proteins [39]. FlgN and FliT interact with FliJ, whereas FliS does not [40]. The cytoplasmic domain of FlhA, one of the export gate component proteins, shows the highest binding affinity for the FlgN/FlgK and FlgN/FlgL complexes, a medium affinity for the FliT/FliD complex, and the lowest affinity for the FliS/FliC complex [41,42,43]. The filament-type proteins are believed to be secreted in the following order: first FlgK, followed by FlgL, FliD, and FliC. Since the filament was constructed on the hook in the IMVs when only supplying the filament-type substrate–chaperone complexes, the affinity of the substrate–chaperone complexes for the export apparatus may determine the export order of the filament-type substrates. However, our results showed that the individual substrates are also secreted in the absence of their predecessors, suggesting that the in vitro export order is not strict for the filamentous type proteins. It might be possible that transcriptional and translational regulation is needed to strictly control the secretion order in vivo.

All the filament-type proteins showed an increase in the export level upon the addition of the FliH_2_/FliI complex in the assay solution (Figure 3). In addition, we found some differences in FliH-I-dependent export level between the filament-type proteins. It has been reported that FlgN and FliT interact with the FliH_2_/FliI complex whereas FliS does not, suggesting that the FliH_2_/FliI complex may be required for more efficient and rapid export of FlgK, FlgL, and FliD than FliC in vivo [39,44,45,46,47]. FlgK, FlgL, and FliD need to be assembled on the hook prior to FliC for filament formation. In the absence of FliH and FliI, a large amount of FliC is leaked into culture media [48], probably due to inefficient assembly of FlgK and FlgL on the hook caused by a failure in the ordered protein export. Therefore, the slight difference in the FliH_2_/FliI complex dependence of export may relate to the export order of the filament-type proteins. 

The export signal of the substrate proteins is found in their disordered N-terminal region, although no significant common sequence has been identified in the region [49]. It has also been reported that the untranslated region of mRNA around the start codon of the substrates is also involved in substrate targeting to the export apparatus [50]. Our in vitro experiments, however, showed that the normal hook–filament structure was successfully constructed inside the IMVs in solution containing no mRNA. Thus, the substrate targeting signal of mRNA is not required for the export of the proteins necessary for hook–filament formation.

The flagellar export apparatus belongs to the type III secretion system family and shares high similarity with the bacterial pathogenic injectisome in its sequence, structure, and function. The injectisome serves to deliver virulence proteins called effectors into their host cells for their infection. The injectisome first exports the needle component proteins and switches the substrate specificity to deliver the effectors after completion of the needle part, just like the flagellum. Therefore, the IMV-based approach we presented here would be useful for studying virulence injectisomes as well.

## 5. Conclusions

We carried out in vitro flagellar protein transport assay to investigate the export process of the filament-type proteins and their assembly into the flagellar filament. The filament-type proteins were transported into the IMVs and formed the filament on the hook–basal body. The hook–filament structures were successfully formed inside the IMVs when all filament-type proteins, their cognate chaperone, FlgD, FlgE, FliK, the ATPase components, and ATP–Mg were simultaneously added into the assay solution. These results indicate that the coordinated flagellar construction is regulated only by the interactions between the flagellar protein export apparatus, the export substrate proteins, and their cognate chaperones.

## Figures and Tables

**Table 1:** Bacterial strains and plasmids.

Strain or Plasmid	Genotype or Description	Reference
*E. coli* strains		
DH5α	F^−^, *mcrA*, Δ(*mrr-hsdRMS-mcrBC*), Φ80d*lacZ*ΔM15, Δ*lacX74*, *deoR*, *recA*1, *araD139*, Δ(*ara leu*)7697, *galU*, *galK*, λ^−^, *rpsL*, *endA*1,*nupG*	
BL21(DE3)	F^−^ *ompT hsd*S_B_ (r_B_^−^ m_B_^−^) *gal dcm* (DE3)	Novagen
*Salmonella* strains		
STH001	∆*flhB* ∆*flgD* ∆*fliT*	[29]
Plasmids		
pBAD33SD	Cm^r^, pBAD33-based vector substituted NheI and EcoRI sites (GCTAGCGAATTC) into SD sequence (GCAGGAGGATTC)	[29]
pTrc99a	Amp^r^, P_trc_ expression vector	
pTrc99aNde	pTrc99a-based vector substituted NcoI sites (cagACCATGgaa) into NdeI sequence (cagCATATGgaa)	This study
pET3c	Amp^r^, T7 expression vector	Novagen
pET15b	Amp^r^, T7 expression vector	Novagen
pITH103	pBAD33SD-*flhB* + *flhDC* (FlhB, FlhD/FlhC)	[29]
pITH105	pET15b-*flgD* (His-FlgD)	[29]
pITH106	pET15b-*flgE* (His-FlgE)	[29]
pMMIJ001	pET15b-*fliJ* (His-FliJ)	[35]
pMKM1702iH	pTrc99a-*his-fliI* + *fliH* (FliH/His-FliI)*, his-fliI* was derived from the pET19b-based plasmid, pMM1901.	[36]
pITH107	pET15b-*fliK* (His-FliK)	[29]
pITH108	pET3c-*fliS* (FliS), the *fliS* gene amplified by PCR was cloned into the NdeI and BamHI sites of the pET3c vector	This study
pITH109	pET15b-*fliC* (His-FliC), the *fliC* gene amplified by PCR was inserted into the NdeI and BamHI sites of pET15b	This study
pITH110	pTrc99aNde-*fliS* + *his-fliC* (His-FliC/FliS), *fliS* derived from pITH108 and *his-fliC* derived from pITH109 were inserted into the NdeI and BamHI sites, and the XbaI and HindIII sites of pTrc99aNde, respectively	This study
pITH111	pET3c-*fliT* (FliT), the *fliT* gene amplified by PCR was cloned into the NdeI and BamHI sites of pET3c	This study
pITH112	pET15b-*fliD* (His-FliD), the *fliD* gene amplified by PCR was inserted into the NdeI and BamHI sites of pET15b	This study
pITH113	pTrc99aNde-*fliT* + *his-fliD* (His-FliD/FliT), *fliT* derived from pITH111 and *his-fliD* derived from pITH112 were subcloned into the NdeI and BamHI sites and the XbaI and HindIII sites of pTrc99aNde, respectively	This study
pMMGN110	pET22b-*flgN* (FlgN), The *flgN* gene amplified by PCR was cloned into the NdeI and BamHI sites of pET22b	[37]
pMMGN300	pET19b-*flgN* (His-FlgN), *flgN* derived from pMMGN110 was subcloned into the NdeI and BamHI sites of pET19b	This study
pMMGK130	pET15b-*flgK* (His-FlgK), the *flgK* gene amplified by PCR was inserted into the NdeI and BamHI sites of pET15b	[37]
pITH116	pET15b-*flgL* (His-FlgL), the *flgL* amplified by PCR was inserted into the NdeI and BamHI sites of pET15b	This study
pITH117	pTrc99aNde-*flgN* + *his-flgK* (His-FlgK/FlgN)*, flgN* derived from pMMGN300 and *his-flgK* derived from pMMGK130 were subcloned into the NdeI and BamHI sites and the XbaI and HindIII sites of pTrc99aNde, respectively	This study
pITH118	pTrc99aNde-*flgN* + *his-flgL* (His-FlgL/FlgN), *flgN* derived from pMMGN300 and *his-flgL* derived from pITH116 were subcloned into the NdeI and BamHI sites and XbaI and HindIII sites of pTrc99aNde, respectively	This study

Amp^r^, ampicillin-resistant; Cm^r^, chloramphenicol-resistant.

## Supplementary Materials

The following are available online at https://www.mdpi.com/2218-273X/10/1/126/s1, Table S1: Primer list for this study. Figure S1: SDS-PAGE analysis of purified proteins used in the in this study. Figure S2: Length distribution of the filament formed on the hook in the IMVs. Figure S3: Magnified electron microscopic image of the hook-filament junction region of the hook-filament complex constructed in the IMV.
