# Supplementary material for: In Vitro Autonomous Construction of the Flagellar Axial Structure in Inverted Membrane Vesicles
Source: Biomolecules. 2020 Jan 11;10(1):126. doi: 10.3390/biom10010126 (PMC7022808; doi:10.3390/biom10010126)
Supplement: Supplementary file 1 [file biomolecules-10-00126-s001.pdf]

Table S1. Primer list for this study.

primer	sequence
pTrc99a-Nde1(+)	acacaggaaacagcatatggaattcgagct
pTrc99a-Nde1(-)	agctcgaattccatatgctgtttcctgtgt
Nde1-fliS(+)	gggaattccatatgtacaccgcgagcggtatcaaagcttat
BamH1-fliS(-)	cgcggatccttaacgagactcctggaaagatgctttcgg
Nde1-fliC(+)	gggaattccatatggcacaagtcattaatacaaacagcctg
BamH1-fliC(-)	cgcggatccttaacgcagtaaagagaggacgttttgcgg
Nde1-fliT(+)	gggaattccatatgacctcaaccgtggagtttatcaaccgt
BamH1-fliT(-)	cgcggatccttatgaggcgccaggcgcatctggcacgag
Nde1-fliD(+)	gggaattccatatggcttcaatttcatcattaggtgtggga
BamH1-fliD(-)	cgcggatcctcaggacttgttcatagctgtaaattgctg
Nde1-flgL(+)	gggaattccatatgcgtatcagtacccagatgatgtacgaa
BamH1-flgL(-)	cgcggatccttaccggttcaactggaaaagcgacattcc

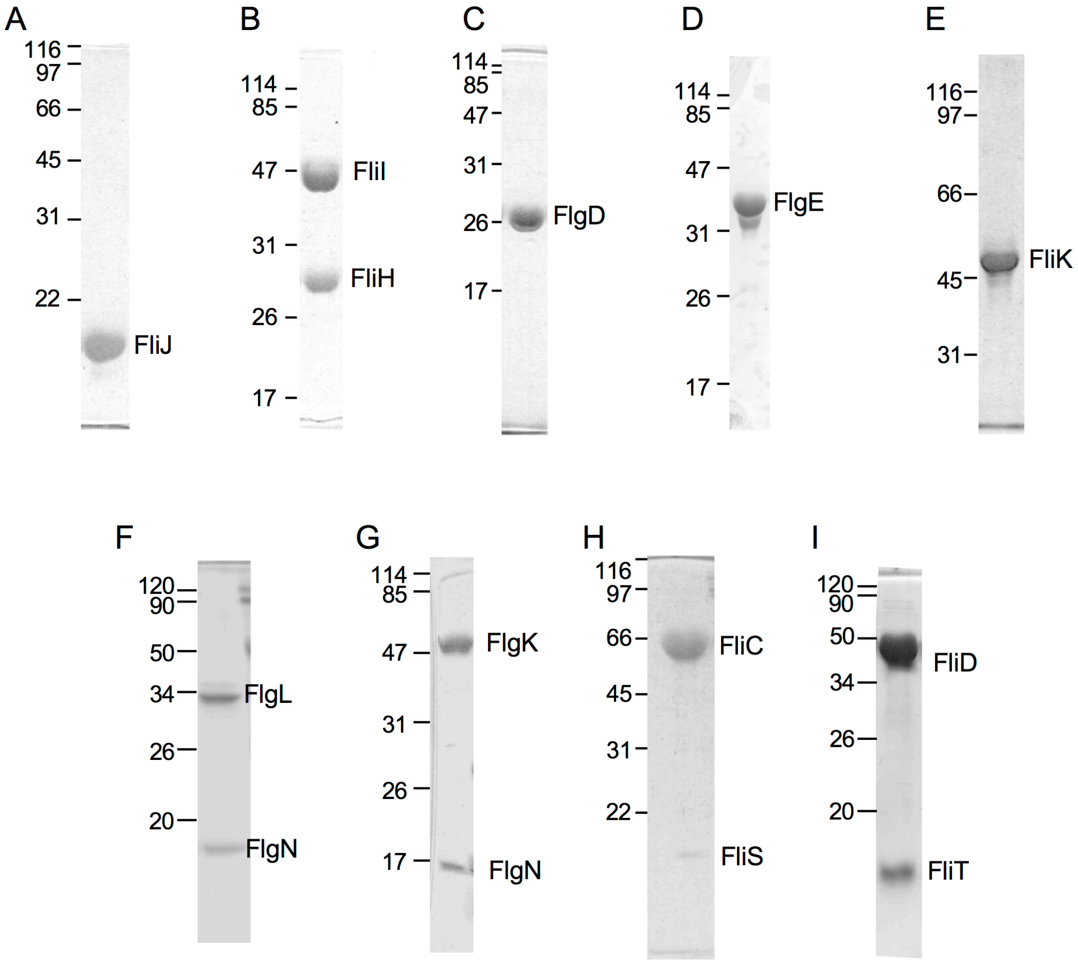

Figure S1. SDS-PAGE analysis of purified proteins used in the in this study. (A) FliJ, (B) the FliH/FliI complex, (C) FlgD, (D) FlgE, (E) FliK, (F) the FlgL/FlgN complex, (G) the FlgK/FlgN complex, (H) the FliC/FliS complex and (I) the FliD/FliT complex.

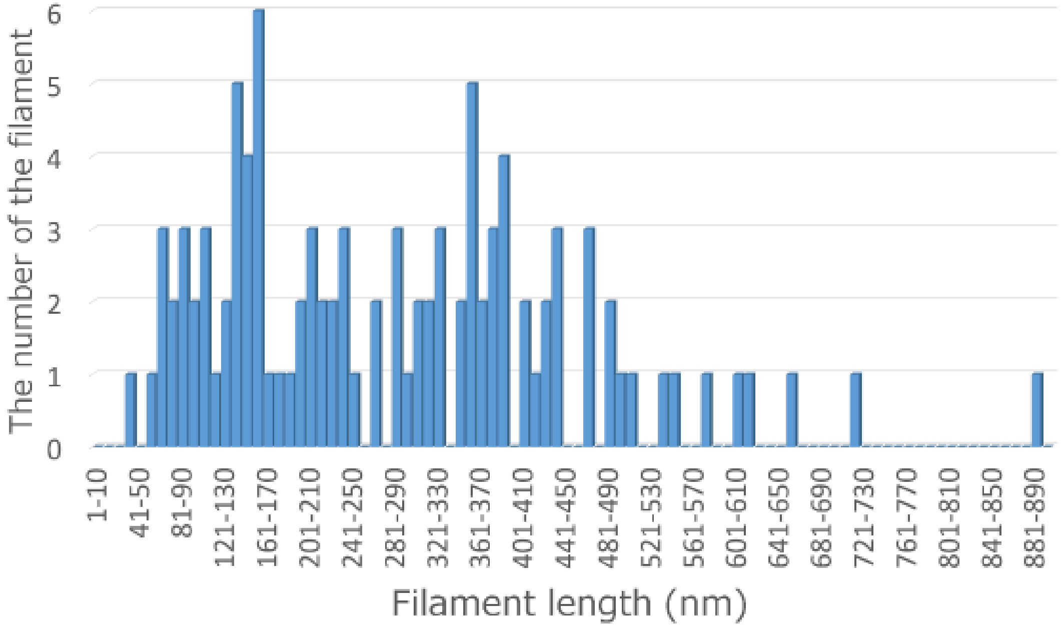

Figure S2. Length distribution of the filament formed on the hook in the IMVs.

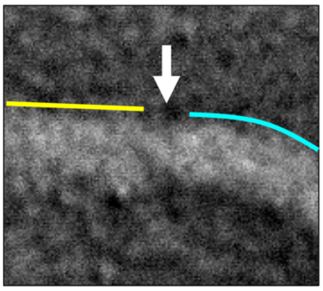

Figure S3. Magnified electron microscopic image of the hook-filament junction region of the hook-filament complex constructed in the IMV. The filament and the hook are indicated by yellow and blue lines, respectively, the hook filament junction is indicated by white arrow.
